# NLRP3 inflammasome in Alzheimer’s disease: molecular mechanisms and emerging therapies

**DOI:** 10.3389/fimmu.2025.1583886

**Published:** 2025-04-07

**Authors:** Zhitao Li, Chunrong Gong

**Affiliations:** ^1^ First School of Clinical Medicine, Shandong University of Traditional Chinese Medicine, Jinan, China; ^2^ Department of Rehabilitation Medicine, Linyi People’s Hospital, Linyi, China

**Keywords:** NLRP3, neuro-inflammation, IL-1β, inflammasome, Alzheimer’s disease, microglia, inhibitor

## Abstract

Alzheimer’s disease (AD) is a progressive neurodegenerative disorder characterized by cognitive decline, memory impairment, and neuroinflammation, with no definitive cure currently available. The NLRP3 inflammasome, a key mediator of neuroinflammation, has emerged as a critical player in AD pathogenesis, contributing to the accumulation of β-amyloid (Aβ) plaques, tau hyperphosphorylation, and neuronal damage. This review explores the mechanisms by which the NLRP3 inflammasome is activated in AD, including its interactions with Aβ, tau, reactive oxygen species (ROS), and pyroptosis. Additionally, it highlights the role of the ubiquitin system, ion channels, autophagy, and gut microbiota in regulating NLRP3 activation. Therapeutic strategies targeting the NLRP3 inflammasome, such as IL-1β inhibitors, natural compounds, and novel small molecules, are discussed as promising approaches to mitigate neuroinflammation and slow AD progression. This review underscores the potential of NLRP3 inflammasome inhibition as a therapeutic avenue for AD.

## 1 Introduction

Alzheimer’s disease (AD) is a prevalent, chronic, and progressive neurodegenerative disorder, often leading to significant cognitive decline and memory impairment in its early stages, with later phases causing substantial daily functioning challenges and psychiatric symptoms (1). Currently, there are no definitive strategies for the prevention or treatment of AD (2), and its incidence is escalating rapidly due to global aging trends (3). The pathogenesis of AD is primarily associated with the accumulation of β-amyloid (Aβ), hyperphosphorylation of tau proteins, and the formation of neurofibrillary tangles. Recent studies have further underscored the critical role of neuroinflammation in the progression of AD (4, 5).

Among the various neuroinflammatory mediators, the NLRP3 inflammasome has gained attention as a potential therapeutic target (6). First described by Martinon et al. (7), the inflammasome is a multi-protein complex that includes caspases, ASC, and cytoplasmic pattern recognition receptors (8). It recognizes pathogen- or danger-associated molecular patterns, activating Caspase-1, which processes IL-1β and IL-18 precursors into active cytokines (9–11). NLRP3, the most extensively studied inflammasome, has been implicated in the pathogenesis of AD (10, 12) This review summarizes the structure, activation mechanisms, and involvement of the NLRP3 inflammasome in AD, as well as potential therapeutic strategies targeting this inflammasome.

## 2 The role of NLRP3 inflammasome in Alzheimer’s disease

### 2.1 Aβ, microglia, and NLRP3 activation

In brain regions affected by AD, microglia are frequently observed in proximity to Aβ plaques. Research has demonstrated that these microglial cells play a crucial role in eliminating Aβ through mechanisms such as phagocytosis and proteolysis (13). The activation of the NLRP3 inflammasome, however, is not solely triggered by fibrillar Aβ but also by smaller Aβ oligomers and protofibrils (14). Aβ induces microglial activation through multiple signaling pathways, including NF-κB, which upregulates the expression of NLRP3 and pro-IL-1β (15). Moreover, soluble Aβ disrupts lysosomal stability, leading to the release of cathepsins and subsequent activation of the NLRP3 inflammasome (16). Additionally, Aβ oligomers impair mitochondrial function, causing oxidative stress and the release of mitochondrial DNA, which further exacerbates NLRP3 inflammasome activation (17). As AD progresses, chronic NLRP3 activation leads to excessive microglial activation, diminishing their ability to clear Aβ and fostering its accumulation (18, 19). Notably, APP/PS1 transgenic mice lacking NLRP3 exhibit reduced Aβ deposition, suggesting that NLRP3 contributes to Aβ accumulation and accelerates disease progression (20).

### 2.2 Tau-induced NLRP3 inflammasome activation

Tau proteins activate the NLRP3 inflammasome in microglial cells via an ASC-dependent mechanism, triggering IL-1β release and neuroinflammation (16, 21). This activation also regulates tau phosphorylation by modulating kinases and phosphatases, leading to tau hyperphosphorylation and aggregation in neurons (22). Hyperphosphorylated tau aggregates (NFTs) are recognized by microglial TLRs, inducing NLRP3 inflammasome assembly through the NF-κB pathway. Additionally, tau uptake by microglial lysosomes can release cathepsin B, further activating the inflammasome. The resulting IL-1β and IL-18 release exacerbates neuroinflammation and accelerates AD (23). Tau precursors can also trigger neuroinflammation and impair memory through NLRP3 pathways (22). Thus, targeting NLRP3 activation may offer a potential AD therapy.

### 2.3 ROS and NLRP3 inflammasome

ROS are crucial regulators of the NLRP3 inflammasome, with their generation closely linked to enzymes such as peroxiredoxin and NADPH oxidase (24). In normal conditions, thioredoxin (TRX) and its binding partner thioredoxin-interacting protein (TXNIP) form a stable complex. However, under oxidative stress, an increase in ROS levels leads to the oxidation of TRX, which in turn neutralizes ROS and causes the dissociation of the TRX-TXNIP complex (25). This dissociation allows TXNIP to interact with NLRP3, thereby recruiting ASC and procaspase-1, which ultimately promotes the assembly and activation of the inflammasome (26). Furthermore, mitochondrial dysfunction can lead to the release of mitochondrial DNA, a potent activator of the NLRP3 inflammasome (27). Different ROS sources selectively modulate NLRP3 signaling in AD (28). Mitochondrial ROS from electron transport chain (ETC) dysfunction exacerbates oxidative damage, impairing neuronal energy metabolism and enhancing NLRP3 activation (29). Conversely, NADPH oxidase-derived ROS, particularly NOX2 in microglia, amplifies ROS production, potentiating NLRP3 signaling and neuroinflammation (30). Targeting these distinct ROS pathways is crucial for precise NLRP3 inhibition in AD.

### 2.4 Pyroptosis mediated by NLRP3 inflammasome

Pyroptosis, an inflammatory programmed cell death, is activated by the NLRP3 inflammasome in AD (31). It promotes pro-inflammatory cytokine secretion, aiding Aβ plaque clearance but also inducing chronic neuroinflammation that accelerates AD progression (32). This process is mediated by gasdermin D (GSDMD) cleavage and caspase-1 activation (33). Pyroptosis occurs via two pathways: (1) The Classical Pathway, where caspase-1 cleaves IL-1β and IL-18 precursors, producing mature cytokines, and modifies GSDMD to form membrane pores, releasing cytokines and driving pyroptosis; and (2) The Non-classical Pathway, where caspase-11 (mice) or caspase-4/5 (humans) is activated by cytosolic LPS, triggering pyroptosis through specific cleavage (34, 35). In AD, Aβ-induced pyroptosis is mediated by the NLRP3-caspase-1-GSDMD axis, exacerbating neuroinflammation and neuronal damage (36–38). GSDMD knockout inhibits astrocyte pyroptosis, mitigating Aβ42-induced brain and vascular damage in APP/PS1 mice (39). These findings suggest that targeting inflammasome-driven pyroptosis holds potential as a novel therapeutic approach for AD.

### 2.5 Integration of Aβ, Tau, ROS, and Autophagy Pathways

**Figure 1:**
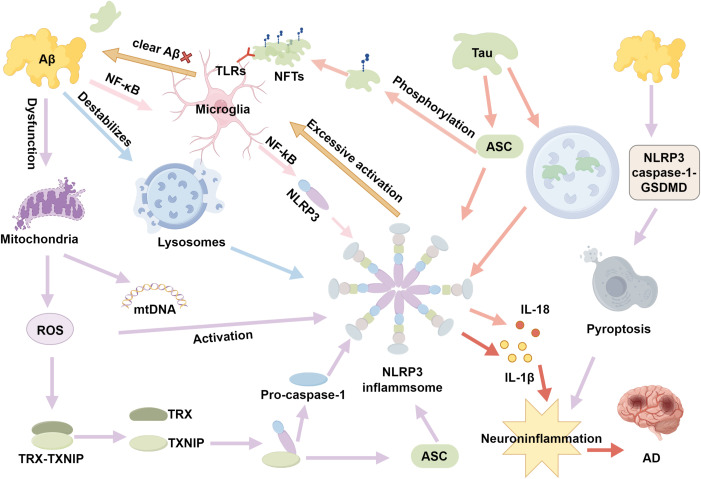
The role of NLRP3 inflammasome in AD.

Recent studies highlight the interconnected mechanisms of Aβ accumulation, tau hyperphosphorylation, ROS generation, and defective autophagy in driving AD progression (34). Aβ and tau aggregates activate microglia, increasing ROS and NLRP3 inflammasome activation, which exacerbates mitochondrial dysfunction and impairs autophagy (40). This creates a vicious cycle in which NLRP3 serves as a pivotal intersection point, amplifying neuroinflammation and neuronal damage (40). Targeting NLRP3 offers a promising therapeutic strategy to disrupt these cascades, addressing the complex interplay of Aβ, tau, ROS, and autophagy in AD (**Figure 1**).

## 3 Mechanisms of NLRP3 inflammasome promoting AD progress

### 3.1 Ubiquitin system

Ubiquitin, a small protein present in all eukaryotic cells, is capable of forming chains through enzymatic processes, which signal target proteins for degradation (41). This modification, referred to as ubiquitination, is carried out by a series of specialized enzymes that alter target proteins, marking them for proteasomal recognition and breakdown (42). In conjunction with autophagy, this system plays a critical role in maintaining cellular homeostasis (43). Disruptions in the ubiquitin-proteasome pathway have been linked to Alzheimer’s disease and other neurodegenerative conditions (44). Within the E1, E2, and E3 enzyme families, E3 ligases such as SCF-FBXL2 selectively target NLRP3 and its associated molecules, including ASC and caspase-1, promoting NLRP3 ubiquitination and proteasomal degradation, thereby regulating its activation (45). Additionally, Cullin1 associates with NLRP3, enhancing its ubiquitination but without triggering degradation, which serves to inhibit NLRP3 inflammasome activation (46).

### 3.2 Ion channels

Ion channels play a critical role in modulating the activation of the NLRP3 inflammasome, with K^+^ efflux identified as a pivotal signaling event (47). In AD, imbalances in the homeostasis of Na^+^ and K^+^ disrupt the electrophysiological properties of neurons, contributing to the pathophysiological change characteristic of the disease (48). Studies by Gritsenko A et al. (49) demonstrated that K^+^ efflux in human monocytes leads to the aggregation of ASC, cleavage of caspase-1, and subsequent processing of GSDMD. Inflammatory factors significantly influence the efficacy of therapies for inflammatory diseases (50–55). Inhibition of NLRP3 or genetic deletion of NLRP3 and GSDMD blocks the release of IL-18, highlighting the crucial role of early inflammasome assembly before IL-1β production (56). Additionally, extracellular ATP, released in response to bacterial toxins, activates P2X7 purinergic receptors, which disturb intracellular ion balance and promote K^+^ efflux (57). This perturbation enables the assembly of NLRP3 with ASC, forming an active inflammasome complex that cleaves procaspase-1 into active caspase-1. Caspase-1 then facilitates the secretion of IL-1β and IL-18 (58). Furthermore, GSDMD is cleaved by the inflammasome, creating membrane pores that allow the release of these cytokines (45).

### 3.3 Autophagosomes and lysosomes

Autophagy is a fundamental cellular process in eukaryotic cells that degrades and recycles cytoplasmic components, such as damaged organelles and protein aggregates, to maintain cellular homeostasis (59). Wang D et al. (60) revealed that excessive accumulation of manganese compromises lysosomal integrity by altering their structure and impairing their function. In manganese-induced NLRP3-caspase-1 inflammasome activation, the release of cathepsin B from lysosomes plays a critical role (61). Additionally, inflammatory stimuli such as alum, crystalline materials, and protein aggregates can trigger autophagy (62), leading to lysosomal destabilization and rupture (63). Following lysosomal rupture, cathepsin B is released and directly interacts with NLRP3, thereby promoting the activation of the NLRP3 inflammasome (62).

### 3.4 ROS production

In AD pathogenesis, Aβ peptides compromise synaptic plasticity and inhibit long-term potentiation (64). Parajuli et al. showed that Aβ promotes the conversion of pro-IL-1β into its active form, IL-1β, thereby enhancing microglia-mediated neurotoxicity (65). This process is largely driven by increased caspase-1 activity and the activation of NOD-like receptors, specifically NLRP3, which features a pyrin domain (66). Mitochondrial-derived ROS, and to a lesser degree, ROS generated by NADPH oxidase, play a pivotal role in initiating NLRP3 activation (67). Elevated ROS levels activate TRPM2 channels, which subsequently activate NLRP3 and caspase-1, thereby increasing IL-1β production (68). Notably, the use of mitochondrial ROS inhibitors, such as DPI, significantly reduces both ROS and IL-1β levels, indicating a suppression of NLRP3 activation (69).

### 3.5 Gut microbiota

GM, a consortium of symbiotic microorganisms within the human intestinal tract, has been implicated in various diseases, including AD. Dysbiosis, or disruptions in microbial composition, is frequently observed in individuals with AD (70). The gut-brain axis, an increasingly studied area, highlights how microbiota influences brain function, with both probiotics and prebiotics playing roles in modulating microbial and immune systems (71). Dysbiosis impairs the intestinal barrier, allowing pathogen-associated molecular patterns (PAMPs) to trigger the release of pro-inflammatory cytokines. These cytokines can then travel to the brain, aggravating the progression of AD (72–74). Moreover, microbial activation of the NLRP3 inflammasome leads to the upregulation of caspase-1 in the AD brain, further advancing disease pathology (75). While short-chain fatty acids (SCFAs) from commensal bacteria bind GPCRs (GPR43, GPR41), inhibit HDACs, reduce cytokines, and downregulate NLRP3 in microglia/astrocytes (76). Thus, restoring a balanced gut microbiota may reduce neuroinflammation and enhance cognitive function in AD patients (77).

## 4 Therapeutic strategies targeting the NLRP3 inflammasome

### 4.1 IL-1β modulation in NLRP3-targeted Alzheimer’s therapy

Early therapeutic strategies targeting the NLRP3 inflammasome pathway have largely focused on IL-1β modulation. Notable agents with proven efficacy include anakinra, a recombinant IL-1 receptor antagonist; canakinumab, a monoclonal antibody against IL-1β; and rilonacept, a soluble decoy receptor that binds IL-1β by incorporating IL-1R1 and IL-1RAcP domains (78). In preclinical experiments using the 3xTg AD model, anakinra was shown to reduce Aβ and tau accumulation, decrease IL-1β levels, and enhance cognitive function (79). Furthermore, both anakinra treatment and genetic deletion of IL-1R improved mitochondrial dysfunction and alleviated memory deficits associated with Aβ in *in vivo* and *in vitro* models (80). Despite these promising results, the challenge of the blood-brain barrier (BBB) has hindered further exploration of IL-1β-based therapies in AD (80). A Phase 2 clinical trial assessing the efficacy of canakinumab in AD patients is currently ongoing (NCT04795466). However, since IL-1β acts as a downstream effector, directly targeting NLRP3 or its inflammasome components could potentially offer more substantial therapeutic advantages (81).

### 4.2 Ginkgolide B and sulforaphane

GB has demonstrated protective effects against ischemic brain injury and neurotoxicity induced by Aβ (82). In models of hypoxic-ischemic brain damage in rats, GB diminishes NLRP3 inflammasome activation, thereby alleviating neuroinflammation and mitigating AD-related pathology in BV2 cells (83). Additionally, GB treatment has been reported to reduce Aβ-induced pathological alterations and inhibit NLRP3 inflammasome activation (84). Furthermore, GB promotes the upregulation of anti-inflammatory markers in M2 microglia, while concurrently suppressing the release of pro-inflammatory cytokines in M1 microglia (85). Through autophagy-dependent pathways, GB also curbs NLRP3 inflammasome activation, ultimately safeguarding cognitive function in SAMP8 mice (84, 86).

SFN exhibits notable anti-inflammatory, antioxidant, and neuroprotective properties (87). Studies suggest that SFN effectively diminishes the release of IL-1β and IL-18 in LPS-activated microglia, while also inhibiting the overexpression of NLRP3 and caspase-1 proteins (88). Furthermore, SFN prevents pyroptosis in microglia by inhibiting caspase-1 activity (89), and attenuates NLRP3 inflammasome activation via the downregulation of NF-κB (88), thus reducing inflammatory responses (90).

### 4.3 Dapansutrile (OLT1177) and MCC950

OLT1177, an orally available and selective inhibitor of the NLRP3 inflammasome, has demonstrated considerable therapeutic promise (91). This compound binds directly to NLRP3, blocking its ATPase function and disrupting several inflammasome activation pathways. In APP/PS1 transgenic mice, OLT1177 treatment partially alleviated cognitive impairments as assessed by the Morris water maze test. It also decreased microglial activation and lowered cortical plaque accumulation (92). Although there is limited research on its application in neurodegenerative disorders, OLT1177’s excellent safety profile, favorable pharmacokinetic characteristics, and minimal side effects underscore its potential as a therapeutic agent for AD (93).

MCC950 is a potent anti-inflammatory compound that selectively inhibits NLRP3 inflammasome activation by targeting its NACHT domain (94). In models of Alzheimer’s disease, MCC950 effectively dampens the inflammasome activation triggered by Aβ or tau, preventing the cleavage and release of caspase-1 and IL-1β. Additionally, it completely halts immune responses induced by Aβ aggregates and low-molecular-weight oligomers (95). *In vitro* experiments using Aβ-stimulated human primary neurons pre-treated with MCC950 demonstrated its capacity to inhibit pyroptosis, thereby significantly reducing Aβ-induced neuronal toxicity. Other NLRP3 inhibitors currently under exploration include IFM-514 (96), CY-09 (97), DFV890 (98), Tranilast (99), Oridonin (100), Selnolast (101), and Inzomelid (102).

### 4.4 Other therapeutic strategies targeting the NLRP3 inflammasome

Nonsteroidal anti-inflammatory drugs (NSAIDs) have shown potential in delaying the onset of Alzheimer’s disease (AD) or reducing its risk, likely through their modulation of the NLRP3 inflammasome pathway (103). For instance, indomethacin, a well-known NSAID, has been demonstrated to inhibit both NLRC4 and NLRP3 inflammasomes. This inhibition leads to a reduction in the expression of IL-1β and caspase-1, thereby alleviating neuroinflammation and mitigating memory deficits associated with AD (104). MicroRNAs have been shown to suppress NLRP3 expression, leading to improved cognitive function in rodent models of Alzheimer’s disease (105, 106). In AD patients, reduced miR-22 levels in AD patients are associated with increased NLRP3 activation, while overexpression of miR-22 inhibits GSDMD-mediated pyroptosis, thereby reducing neuroinflammation and cognitive decline in AD mice (107). These findings highlight the potential of targeting miRNAs to modulate NLRP3 activity as a novel therapeutic strategy for AD.

In addition to miRNAs, autophagy and mitophagy also modulate NLRP3 activation. For example, IIIM-941 has been shown to induce autophagy via AMPK, inhibiting NLRP3 activity (108). A-68930 activates dopamine D1 receptors, promoting NLRP3 degradation through AMPK/autophagy, reducing IL-1β/IL-18 secretion, and mitigating Aβ1-42-induced neuroinflammation (109). Ginkgo biloba extract EGb 761 enhances microglial autophagy, downregulates NLRP3, and attenuates Aβ-induced IL-1β/caspase-1 activation in TgCRND8 mice (110). Furthermore, Quercetin stimulates mitophagy, suppressing mtROS-driven NLRP3 activation and protecting against neuronal damage (111). These studies suggest that enhancing autophagy and mitophagy in microglia may offer a promising therapeutic approach for AD.

### 4.8 New therapies focus on the NLRP3 inflammasome

A recent study has identified cornuside as a promising anti-AD agent. Cornuside has been shown to restores mitophagic flux, enabling the efficient the removal of damaged mitochondria and the recovery of mitochondrial function. These mechanisms contribute to the inhibition of NLRP3 inflammasome activation, thereby reducing neuronal and synaptic damage and improving cognitive function (112). Structurally unique diterpenoids, isolated from the mangrove plant *Excoecaria agallocha L.*, have emerged as promising anti-neuroinflammatory agents. These compounds exert their effects by inhibiting macrophage polarization and suppressing the activation of the NLRP3 inflammasome, highlighting their potential in mitigating neuroinflammation (113). Additionally, Thonningianin A (ThA) has demonstrated the ability to suppress NLRP3 inflammasome-driven inflammation and curb the overactivation of microglia and astrocytes through the induction of autophagy (114). Moreover, autophagy has been implicated in mitigating neuroinflammation in AD by modulating NLRP3 inflammasome activity (115). Consequently, targeting the autophagy-NLRP3 inflammasome axis using ThA holds potential as a novel therapeutic approach for AD (23). Additionally, research suggests that Eriodictyol exerts beneficial effects on AD by inhibiting NLRP3 activation. Eriodictyol can cross the BBB and significantly reduce the expression of NLRP3, caspase-1, and ASC proteins in brain tissue, while also decreasing the inflammatory cytokines IL-1β and IL-18. These effects improve cognitive function and memory (116), as well as attenuate AD pathology (23, 117). The ongoing phase 3 trial TRAILBLAZER-ALZ 2 (NCT04437511) (118), phase 2 clinical trial (NCT04795466) (81) and other AD clinical trials will offer a more comprehensive strategy for AD treatment (119) (**Table 1**).

**Table 1:** The therapies targeting NLRP3 Inflammasome in AD.

Therapeutic Strategy	Mechanism	Effect
Targeting IL-1β (Anakinra)	Modulates IL-1β activity by using IL-1 receptor antagonists, monoclonal antibodies, and decoy receptors.	Reduce IL-1β, improve cognitive function, and alleviate neuroinflammation.
Ginkgolide B (GB)	Inhibits NLRP3 inflammasome activation and reduces neuroinflammation.	GB reduces Aβ-induced pathology, enhances M2 microglia, suppresses pro-inflammatory cytokines, and improves cognitive function.
Sulforaphane (SFN)	Inhibits NLRP3 inflammasome activation via NF-κB downregulation and reduces pyroptosis.	SFN diminishes IL-1β/IL-18 release, inhibits caspase-1, and reduces NLRP3 overexpression in LPS-activated microglia. Shows anti-inflammatory and neuroprotective effects.
Dapansutrile (OLT1177)	Selectively inhibits NLRP3 inflammasome by blocking ATPase function and inflammasome activation.	OLT1177 improves cognitive function, reduces microglial activation, and lowers Aβ plaque accumulation in AD mouse models. Favorable pharmacokinetic properties.
MCC950	Selectively inhibits NLRP3 inflammasome via NACHT domain targeting.	MCC950 inhibits inflammasome activation by Aβ/tau, reducing IL-1β release and preventing neuronal toxicity in AD models.
Ketone Bodies	Ketogenic diets and ketone bodies (e.g., β-hydroxybutyrate) inhibit NLRP3 inflammasome activation and reduce Aβ buildup.	β-Hydroxybutyrate inhibits NLRP3 inflammasome, reduces Aβ internalization, and mitigates AD progression. 2-DG enhances bioenergetic capacity and promotes Aβ clearance.
Other Strategies	NSAIDs, microRNAs, autophagy, mitophagy, and botanical extracts modulate NLRP3 inflammasome activity.	Indomethacin, miR-138–5p, miR-223, Quercetin, Ginkgo biloba, and others reduce NLRP3 activation, improving cognition and reducing neuroinflammation.
New Therapies	Targets autophagy, mitophagy, and inflammasome activation.	Cornuside, Thonningianin A, and Eriodictyol inhibit NLRP3 inflammasome activation, promote mitophagic flux, and improve cognitive function in AD models.

## 5 Conclusion

The NLRP3 inflammasome plays a pivotal role in the pathogenesis of Alzheimer’s disease by driving neuroinflammation, Aβ accumulation, tau pathology, and neuronal damage. Its activation is influenced by multiple factors, including ROS, mitochondrial dysfunction, and gut microbiota dysbiosis. Targeting the NLRP3 inflammasome through various therapeutic strategies, such as IL-1β modulation, natural compounds, and small-molecule inhibitors, offers promising potential to alleviate neuroinflammation and slow disease progression. Future research should focus on developing NLRP3-targeted therapies that can effectively cross the blood-brain barrier and provide long-term benefits in AD patients. Moreover, integrating biomarker identification and precision-targeted drug design into clinical research could expedite the transition from bench to bedside, ultimately offering earlier intervention and better protection against neurodegeneration in individuals at risk for or diagnosed with AD. By addressing the central role of the NLRP3 inflammasome in AD, these therapeutic approaches may pave the way for more effective treatments for this debilitating disease.

## References

[1] Furman D Chang J Lartigue L Bolen CR Haddad F Gaudilliere B. Expression of specific inflammasome gene modules stratifies older individuals into two extreme clinical and immunological states. Nat Med. (2017) 23:174–84. doi: 10.1038/nm.4267 PMC5320935 28092664

[2] Bali J Gheinani AH Zurbriggen S Rajendran L. Role of genes linked to sporadic Alzheimer’s disease risk in the production of beta-amyloid peptides. Proc Natl Acad Sci U.S.A. (2012) 109:15307–11. doi: 10.1073/pnas.1201632109 PMC3458335 22949636

[3] Yamazaki Y Zhao N Caulfield TR Liu CC Bu G. Apolipoprotein E and Alzheimer disease: pathobiology and targeting strategies. Nat Rev Neurol. (2019) 15:501–18. doi: 10.1038/s41582-019-0228-7 PMC7055192 31367008

[4] Pallo SP Johnson GV. Tau facilitates Abeta-induced loss of mitochondrial membrane potential independent of cytosolic calcium fluxes in mouse cortical neurons. Neurosci Lett. (2015) 597:32–7. doi: 10.1016/j.neulet.2015.04.021 PMC4442715 25888814

[5] Hansen DV Hanson JE Sheng M. Microglia in alzheimer’s disease. J Cell Biol. (2018) 217:459–72. doi: 10.1083/jcb.201709069 PMC5800817 29196460

[6] Pereira CF Santos AE Moreira PI Pereira AC Sousa FJ Cardoso SM. Is Alzheimer’s disease an inflammasomopathy? Ageing Res Rev. (2019) 56:100966. doi: 10.1016/j.arr.2019.100966 31577960

[7] Martinon F Burns K Tschopp J. The inflammasome: a molecular platform triggering activation of inflammatory caspases and processing of proIL-beta. Mol Cell. (2002) 10:417–26. doi: 10.1016/S1097-2765(02)00599-3 12191486

[8] Xue Y Enosi Tuipulotu Tan WH Kay C Man SM. Emerging activators and regulators of inflammasomes and pyroptosis. Trends Immunol. (2019) 40:1035–52. doi: 10.1016/j.it.2019.09.005 31662274

[9] Swanson KV Deng M Ting JP. The NLRP3 inflammasome: molecular activation and regulation to therapeutics. Nat Rev Immunol. (2019) 19:477–89. doi: 10.1038/s41577-019-0165-0 PMC7807242 31036962

[10] Ozaki E Campbell M Doyle SL. Targeting the NLRP3 inflammasome in chronic inflammatory diseases: current perspectives. J Inflammation Res. (2015) 8:15–27. doi: 10.2147/JIR.S51250 PMC4303395 25653548

[11] Franchi L Eigenbrod T Munoz-Planillo R Ozkurede U Kim YG Arindam C. Cytosolic double-stranded RNA activates the NLRP3 inflammasome via MAVS-induced membrane permeabilization and K+ efflux. J Immunol. (2014) 193:4214–22. doi: 10.4049/jimmunol.1400582 PMC4185247 25225670

[12] Heneka MT McManus RM Latz E. Inflammasome signalling in brain function and neurodegenerative disease. Nat Rev Neurosci. (2018) 19:610–21. doi: 10.1038/s41583-018-0055-7 30206330

[13] Merighi S Nigro M Travagli A Gessi S. Microglia and alzheimer’s disease. Int J Mol Sci. (2022) 23:12990. doi: 10.3390/ijms232112990 36361780 PMC9657945

[14] Bai H Zhang Q. Activation of NLRP3 inflammasome and onset of alzheimer’s disease. Front Immunol. (2021) 12:701282. doi: 10.3389/fimmu.2021.701282 34381452 PMC8350495

[15] Nopparat C Boontor A Kutpruek S Govitrapong P. The role of melatonin in amyloid beta-induced inflammation mediated by inflammasome signaling in neuronal cell lines. Sci Rep. (2023) 13:17841. doi: 10.1038/s41598-023-45220-1 37857668 PMC10587142

[16] Van Zeller M Dias D Sebastiao AM Valente CA. NLRP3 inflammasome: A starring role in amyloid-beta- and tau-driven pathological events in alzheimer’s disease. J Alzheimers Dis. (2021) 83:939–61. doi: 10.3233/JAD-210268 PMC8543248 34366341

[17] Li X Jin Y Ding X Zhu T Wei C Yao L. Long-term exercise training inhibits inflammation by suppressing hippocampal NLRP3 in APP/PS1 mice. Sports Med Health Sci. (2023) 5:329–35. doi: 10.1016/j.smhs.2023.09.009 PMC10831383 38314041

[18] Venegas C Kumar S Franklin BS Dierkes T Brinkschulte R Tejera D. Microglia-derived ASC. specks cross-seed amyloid-beta Alzheimer’s disease. Nat. (2017) 552:355–61. doi: 10.1038/nature25158 29293211

[19] Friker LL Scheiblich H Hochheiser IV Brinkschulte R Riedel D Latz E. beta-amyloid clustering around ASC fibrils boosts its toxicity in microglia. Cell Rep. (2020) 30:3743–3754 e6. doi: 10.1016/j.celrep.2020.02.025 32187546 PMC8729885

[20] Barczuk J Siwecka N Lusa W Rozpędek-Kamińska W Kucharska E Majsterek I. Targeting NLRP3-mediated neuroinflammation in alzheimer’s disease treatment. Int J Mol Sci. (2022) 23:8979. doi: 10.3390/ijms23168979 36012243 PMC9409081

[21] Stancu IC Cremers N Vanrusselt H Couturier J Vanoosthuyse A Kessels S. Aggregated Tau activates NLRP3-ASC inflammasome exacerbating exogenously seeded and non-exogenously seeded Tau pathology *in vivo* . Acta Neuropathol. (2019) 137:599–617. doi: 10.1007/s00401-018-01957-y 30721409 PMC6426830

[22] Ising C Venegas C Zhang S Scheiblich H Schmidt SV Vieira-Saecker A. NLRP3 inflammasome activation drives tau pathology. Nature. (2019) 575:669–73. doi: 10.1038/s41586-019-1769-z PMC7324015 31748742

[23] Zu R Lu H Liu W Shao S Zheng J Ying X. Research progress in the molecular mechanism of NLRP3 inflammasome in alzheimer’s disease and regulation by natural plant products. Mol Neurobiol. (2025). doi: 10.1007/s12035-025-04715-w 39875780

[24] Franchi L Eigenbrod T MuÃ±oz-Planillo R NuÃ±ez G. The inflammasome: a caspase-1-activation platform that regulates immune responses and disease pathogenesis. Nat Immunol. (2009) 10:241–7. doi: 10.1038/ni.1703 PMC2820724 19221555

[25] Qayyum N Haseeb M Kim MS Choi S. Role of thioredoxin-interacting protein in diseases and its therapeutic outlook. Int J Mol Sci. (2021) 22:2754. doi: 10.3390/ijms22052754 33803178 PMC7963165

[26] Zhang Y Xing CJ Liu X Li YH Jia J Feng JG. Thioredoxin-interacting protein (TXNIP) knockdown protects against sepsis-induced brain injury and cognitive decline in mice by suppressing oxidative stress and neuroinflammation. Oxid Med Cell Longev 2022. (2022) p:8645714. doi: 10.1155/2022/8645714 PMC9098358 35571246

[27] Yu JW Lee MS. Mitochondria and the NLRP3 inflammasome: physiological and pathological relevance. Arch Pharm Res. (2016) 39:1503–18. doi: 10.1007/s12272-016-0827-4 27600432

[28] Wei P Yang F Zheng Q Tang W Li JJFiCN. The potential role of the NLRP3 inflammasome activation as a link between mitochondria ROS generation and neuroinflammation in postoperative cognitive dysfunction. Front Cell Neurosci. (2019) 13:73. doi: 10.3389/fncel.2019.00073 30873011 PMC6401615

[29] Billingham LK Stoolman JS Vasan K Rodriguez AE Poor TA Szibor M. Mitochondrial electron transport chain is necessary for NLRP3 inflammasome activation. Nat Immunol. (2022) 23:692–704. doi: 10.1038/s41590-022-01185-3 35484407 PMC9098388

[30] McCarty MF DiNicolantonio JJ Lerner A. A fundamental role for oxidants and intracellular calcium signals in Alzheimer’s pathogenesis—and how a comprehensive antioxidant strategy may aid prevention of this disorder. Int J Mol Sci. (2021) 22:2140. doi: 10.3390/ijms22042140 33669995 PMC7926325

[31] Moonen S Koper MJ Van Schoor E Schaeverbeke JM Vandenberghe R von Arnim CAF. Pyroptosis in Alzheimer’s disease: cell type-specific activation in microglia, astrocytes and neurons. Acta Neuropathol. (2023) 145:175–95. doi: 10.1007/s00401-022-02528-y 36481964

[32] Lee S Cho HJ Ryu JH. Innate immunity and cell death in alzheimer’s disease. ASN Neuro. (2021) 13:17590914211051908. doi: 10.1177/17590914211051908 34668411 PMC8532209

[33] Xue W Cui D Qiu Y. Research progress of pyroptosis in alzheimer’s disease. Front Mol Neurosci. (2022) 15:872471. doi: 10.3389/fnmol.2022.872471 35782390 PMC9244792

[34] Liang T Zhang Y Wu S Chen Q Wang L. The role of NLRP3 inflammasome in alzheimer’s disease and potential therapeutic targets. Front Pharmacol. (2022) 13:845185. doi: 10.3389/fphar.2022.845185 35250595 PMC8889079

[35] Mangalmurti A Lukens JR. How neurons die in Alzheimer’s disease: Implications for neuroinflammation. Curr Opin Neurobiol. (2022) 75:102575. doi: 10.1016/j.conb.2022.102575 35691251 PMC9380082

[36] Han C Yang Y Guan Q Zhang X Shen H Sheng Y. New mechanism of nerve injury in Alzheimer’s disease: beta-amyloid-induced neuronal pyroptosis. J Cell Mol Med. (2020) 24:8078–90. doi: 10.1111/jcmm.v24.14 PMC7348172 32521573

[37] Jia J Zhang X Xu G Zeng X Li L. Thioredoxin-1 inhibits amyloid-beta(25-35)-induced activation of NLRP1/caspase-1/GSDMD pyroptotic pathway in PC12 cells. Mol Biol Rep. (2022) 49:3445–52. doi: 10.1007/s11033-022-07177-8 35072836

[38] Huang Y Li X Luo G Wang J Li R Zhou C. Pyroptosis as a candidate therapeutic target for Alzheimer’s disease. Front Aging Neurosci. (2022) 14:996646. doi: 10.3389/fnagi.2022.996646 36185484 PMC9520296

[39] Hong W Hu C Wang C Zhu B Tian M Qin H. Effects of amyloid beta (Abeta)42 and Gasdermin D on the progression of Alzheimer’s disease *in vitro* and *in vivo* through the regulation of astrocyte pyroptosis. Aging (Albany NY). (2023) 15:12209–24. doi: 10.18632/aging.205174 PMC10683627 37921870

[40] Ayyubova G Madhu LN. Microglial NLRP3 inflammasomes in alzheimer’s disease pathogenesis: from interaction with autophagy/mitophagy to therapeutics. Mol Neurobiol. (2025) p:1–20. doi: 10.1007/s12035-025-04758-z 39951189

[41] Bialek W Collawn JF Bartoszewski R. Ubiquitin-dependent and independent proteasomal degradation in host-pathogen interactions. Molecules. (2023) 28:6740. doi: 10.3390/molecules28186740 37764516 PMC10536765

[42] Finley D Ciechanover A Varshavsky A. Ubiquitin as a central cellular regulator. Cell. (2004) 116:S29–32. doi: 10.1016/S0092-8674(03)00971-1 15055578

[43] Ji CH Kwon YT. Crosstalk and interplay between the ubiquitin-proteasome system and autophagy. Mol Cells. (2017) 40:441–9. doi: 10.14348/molcells.2017.0115 PMC5547213 28743182

[44] Hegde AN Smith SG Duke LM Pourquoi A Vaz S. Perturbations of ubiquitin-proteasome-mediated proteolysis in aging and alzheimer’s disease. Front Aging Neurosci. (2019) 11:324. doi: 10.3389/fnagi.2019.00324 31866849 PMC6910070

[45] Lopez-Castejon G. Control of the inflammasome by the ubiquitin system. FEBS J. (2020) 287:11–26. doi: 10.1111/febs.v287.1 31679183 PMC7138099

[46] Wan P Zhang Q Liu W Jia Y Ai S Wang T. Cullin1 binds and promotes NLRP3 ubiquitination to repress systematic inflammasome activation. FASEB J. (2019) 33:5793–807. doi: 10.1096/fj.201801681R 30653357

[47] Koumangoye R. The role of Cl(-) and K(+) efflux in NLRP3 inflammasome and innate immune response activation. Am J Physiol Cell Physiol. (2022) 322:C645–52. doi: 10.1152/ajpcell.00421.2021 35171697

[48] Graham SF Nasarauddin MB Carey M McGuinness B Holscher C Kehoe PG. Quantitative measurement of [Na+] and [K+] in postmortem human brain tissue indicates disturbances in subjects with Alzheimer’s disease and dementia with Lewy bodies. J Alzheimers Dis. (2015) 44:851–7. doi: 10.3233/JAD-141869 25362038

[49] Gritsenko A Yu S Martin-Sanchez F Diaz-Del-Olmo I Nichols EM Davis DM. Priming is dispensable for NLRP3 inflammasome activation in human monocytes *in vitro* . Front Immunol. (2020) 11:565924. doi: 10.3389/fimmu.2020.565924 33101286 PMC7555430

[50] Zhai X Zhang H Xia Z Liu M Du G Jiang Z. Oxytocin alleviates liver fibrosis via hepatic macrophages. JHEP Rep. (2024) 6:101032. doi: 10.1016/j.jhepr.2024.101032 38882603 PMC11177191

[51] Xiao J Lin H Liu B Xia Z Zhang J Jin J. Decreased S1P and SPHK2 are involved in pancreatic acinar cell injury. biomark Med. (2019) 13:627–37. doi: 10.2217/bmm-2018-0404 31157539

[52] Xiao J Huang K Lin H Xia Z Zhang J Li D. Mogroside II(E) inhibits digestive enzymes via suppression of interleukin 9/interleukin 9 receptor signalling in acute pancreatitis. Front Pharmacol. (2020) 11:859. doi: 10.3389/fphar.2020.00859 32587518 PMC7298197

[53] Zhang H Xia T Xia Z Zhou H Li Z Wang W. KIF18A inactivates hepatic stellate cells and alleviates liver fibrosis through the TTC3/Akt/mTOR pathway. Cell Mol Life Sci. (2024) 81:96. doi: 10.1007/s00018-024-05114-5 38372748 PMC10876760

[54] Zhang JJ Rizk R Li X Lee BG Matthies ML Bietz KA. Interleukin-10 exhibit dose-dependent effects on macrophage phenotypes and cardiac remodeling after myocardial infarction. Front Physiol. (2024) 15:1481460. doi: 10.3389/fphys.2024.1481460 39882328 PMC11774956

[55] Zhang H Ni M Wang H Zhang J Jin D Busuttil RW. Gsk3beta regulates the resolution of liver ischemia/reperfusion injury via MerTK. JCI Insight. (2023) 8:e151819. doi: 10.1172/jci.insight.151819 36422999 PMC9870084

[56] Vande Walle L Stowe IB Sacha P Lee BL Demon D Fossoul A. MCC950/CRID3 potently targets the NACHT domain of wild-type NLRP3 but not disease-associated mutants for inflammasome inhibition. PloS Biol. (2019) 17:e3000354. doi: 10.1371/journal.pbio.3000354 31525186 PMC6762198

[57] Dosch M Gerber J Jebbawi F Beldi G. Mechanisms of ATP release by inflammatory cells. Int J Mol Sci. (2018) 19:1222. doi: 10.3390/ijms19041222 29669994 PMC5979498

[58] Savio LEB de Andrade Mello P da Silva CG Coutinho-Silva R. The P2X7 receptor in inflammatory diseases: angel or demon? Front Pharmacol. (2018) 9:52. doi: 10.3389/fphar.2018.00052 29467654 PMC5808178

[59] Xia Y Liu N Xie X Bi G Ba H Li L. The macrophage-specific V-ATPase subunit ATP6V0D2 restricts inflammasome activation and bacterial infection by facilitating autophagosome-lysosome fusion. Autophagy. (2019) 15:960–75. doi: 10.1080/15548627.2019.1569916 PMC6526827 30681394

[60] Wang D Zhang J Jiang W Cao Z Zhao F Cai T. The role of NLRP3-CASP1 in inflammasome-mediated neuroinflammation and autophagy dysfunction in manganese-induced, hippocampal-dependent impairment of learning and memory ability. Autophagy. (2017) 13:914–27. doi: 10.1080/15548627.2017.1293766 PMC5446056 28318352

[61] Tinkov AA Paoliello MMB Mazilina AN Skalny AV Martins AC Voskresenskaya ON. Molecular targets of manganese-induced neurotoxicity: A five-year update. Int J Mol Sci. (2021) 22:4646. doi: 10.3390/ijms22094646 33925013 PMC8124173

[62] Biasizzo M Kopitar-Jerala N. Interplay between NLRP3 inflammasome and autophagy. Front Immunol. (2020) 11:591803. doi: 10.3389/fimmu.2020.591803 33163006 PMC7583715

[63] Zhou J Li C Lu M Jiang G Chen S Li H. Pharmacological induction of autophagy reduces inflammation in macrophages by degrading immunoproteasome subunits. PloS Biol. (2024) 22:e3002537. doi: 10.1371/journal.pbio.3002537 38447109 PMC10917451

[64] Li S Jin M Koeglsperger T Shepardson NE Shankar GM Selkoe DJ. Soluble Abeta oligomers inhibit long-term potentiation through a mechanism involving excessive activation of extrasynaptic NR2B-containing NMDA receptors. J Neurosci. (2011) 31:6627–38. doi: 10.1523/JNEUROSCI.0203-11.2011 PMC3100898 21543591

[65] Parajuli B Sonobe Y Horiuchi H Takeuchi H Mizuno T Suzumura A. Oligomeric amyloid beta induces IL-1beta processing via production of ROS: implication in Alzheimer’s disease. Cell Death Dis. (2013) 4:e975. doi: 10.1038/cddis.2013.503 24357806 PMC3877570

[66] Platnich JM Muruve DA. NOD-like receptors and inflammasomes: A review of their canonical and non-canonical signaling pathways. Arch Biochem Biophys. (2019) 670:4–14. doi: 10.1016/j.abb.2019.02.008 30772258

[67] Lee H Jose PA. Coordinated contribution of NADPH oxidase- and mitochondria-derived reactive oxygen species in metabolic syndrome and its implication in renal dysfunction. Front Pharmacol. (2021) 12:670076. doi: 10.3389/fphar.2021.670076 34017260 PMC8129499

[68] Zheng Q Tan Q Ren Y Reinach PS Li L Ge C. Hyperosmotic stress-induced TRPM2 channel activation stimulates NLRP3 inflammasome activity in primary human corneal epithelial cells. Invest Ophthalmol Vis Sci. (2018) 59:3259–68. doi: 10.1167/iovs.18-23965 29971445

[69] Aminzadeh M Roghani M Sarfallah A Riazi GH. TRPM2 dependence of ROS-induced NLRP3 activation in Alzheimer’s disease. Int Immunopharmacol. (2018) 54:78–85. doi: 10.1016/j.intimp.2017.10.024 29107864

[70] Mancuso C Santangelo R. Alzheimer’s disease and gut microbiota modifications: The long way between preclinical studies and clinical evidence. Pharmacol Res. (2018) 129:329–36. doi: 10.1016/j.phrs.2017.12.009 29233677

[71] Suganya K Koo BS. Gut-brain axis: role of gut microbiota on neurological disorders and how probiotics/prebiotics beneficially modulate microbial and immune pathways to improve brain functions. Int J Mol Sci. (2020) 21:7551. doi: 10.3390/ijms21207551 33066156 PMC7589356

[72] Jie Z Xia H Zhong SL Feng Q Li S Liang S. The gut microbiome in atherosclerotic cardiovascular disease. Nat Commun. (2017) 8:845. doi: 10.1038/s41467-017-00900-1 29018189 PMC5635030

[73] Askarova S Umbayev B Masoud AR Kaiyrlykyzy A Safarova Y Tsoy A. The links between the gut microbiome, aging, modern lifestyle and alzheimer’s disease. Front Cell Infect Microbiol. (2020) 10:104. doi: 10.3389/fcimb.2020.00104 32257964 PMC7093326

[74] Yamazaki Y Kanekiyo T. Blood-brain barrier dysfunction and the pathogenesis of alzheimer’s disease. Int J Mol Sci. (2017) 18:1965. doi: 10.3390/ijms18091965 28902142 PMC5618614

[75] Pennisi M Crupi R Di Paola R Ontario ML Bella R Calabrese EJ. Inflammasomes, hormesis, and antioxidants in neuroinflammation: Role of NRLP3 in Alzheimer disease. J Neurosci Res. (2017) 95:1360–72. doi: 10.1002/jnr.23986 27862176

[76] Qian X-h Xie R-Y Liu X-l Tang H-dJA. Mechanisms of short-chain fatty acids derived from gut microbiota in Alzheimer’s disease. Aging Dis. (2022) 13:1252–66. doi: 10.14336/AD.2021.1215 PMC9286902 35855330

[77] Shen H Guan Q Zhang X Yuan C Tan Z Zhai L. New mechanism of neuroinflammation in Alzheimer’s disease: The activation of NLRP3 inflammasome mediated by gut microbiota. Prog Neuropsychopharmacol Biol Psychiatry. (2020) 100:109884. doi: 10.1016/j.pnpbp.2020.109884 32032696

[78] Dubois EA Rissmann R Cohen AF. Rilonacept and canakinumab. Br J Clin Pharmacol. (2011) 71:639–41. doi: 10.1111/j.1365-2125.2011.03958.x PMC3093069 21375570

[79] Kitazawa M Cheng D Tsukamoto MR Koike MA Wes PD Vasilevko V. Blocking IL-1 signaling rescues cognition, attenuates tau pathology, and restores neuronal beta-catenin pathway function in an Alzheimer’s disease model. J Immunol. (2011) 187:6539–49. doi: 10.4049/jimmunol.1100620 PMC4072218 22095718

[80] Batista AF Rody T Forny-Germano L Cerdeiro S Bellio M Ferreira ST. Interleukin-1beta mediates alterations in mitochondrial fusion/fission proteins and memory impairment induced by amyloid-beta oligomers. J Neuroinflamm. (2021) 18:54. doi: 10.1186/s12974-021-02099-x PMC7897381 33612100

[81] McManus RM Latz E. NLRP3 inflammasome signalling in Alzheimer’s disease. Neuropharmacology. (2024) 252:109941. doi: 10.1016/j.neuropharm.2024.109941 38565393

[82] Wang J Ding Y Zhuang L Wang Z Xiao W Zhu J. Ginkgolide B−induced AMPK pathway activation protects astrocytes by regulating endoplasmic reticulum stress, oxidative stress and energy metabolism induced by Abeta1−42. Mol Med Rep. (2021) 23:457. doi: 10.3892/mmr.2021.12096 33880582 PMC8072312

[83] Liu GZ Niu TT Yu Q Xu BL Li XQ Yuan BY. Ginkgolide attenuates memory impairment and neuroinflammation by suppressing the NLRP3/caspase-1 pathway in Alzheimer’s disease. Aging (Albany NY). (2023) 15:10237–52. doi: 10.18632/aging.205072 PMC10599747 37793010

[84] Shao L Dong C Geng D He Q Shi Y. Ginkgolide B inactivates the NLRP3 inflammasome by promoting autophagic degradation to improve learning and memory impairment in Alzheimer’s disease. Metab Brain Dis. (2022) 37:329–41. doi: 10.1007/s11011-021-00886-2 35050445

[85] You JE Kim Ej Kim HW Kim JS Kim K Kim PH. Exploring the role of guanylate-binding protein-2 in activated microglia-mediated neuroinflammation and neuronal damage. Biomedicines. (2024) 12:1130. doi: 10.3390/biomedicines12051130 38791092 PMC11117630

[86] Zhang Y Zhao Y Zhang J Gao Y Li S Chang C. Ginkgolide B inhibits NLRP3 inflammasome activation and promotes microglial M2 polarization in Abeta(1-42)-induced microglia cells. Neurosci Lett. (2021) 764:136206. doi: 10.1016/j.neulet.2021.136206 34478813

[87] Kiser C Gonul CP Olcum M Genc S. Inhibitory effects of sulforaphane on NLRP3 inflammasome activation. Mol Immunol. (2021) 140:175–85. doi: 10.1016/j.molimm.2021.10.014 34717147

[88] Brandenburg LO Kipp M Lucius R Pufe T Wruck CJ. Sulforaphane suppresses LPS-induced inflammation in primary rat microglia. Inflammation Res. (2010) 59:443–50. doi: 10.1007/s00011-009-0116-5 19924513

[89] Greaney AJ Maier NK Leppla SH Moayeri M. Sulforaphane inhibits multiple inflammasomes through an Nrf2-independent mechanism. J Leukoc Biol. (2016) 99:189–99. doi: 10.1189/jlb.3A0415-155RR PMC4673482 26269198

[90] Tufekci KU Ercan I Isci KB Olcum M Tastan B Gonul CP. Sulforaphane inhibits NLRP3 inflammasome activation in microglia through Nrf2-mediated miRNA alteration. Immunol Lett. (2021) 233:20–30. doi: 10.1016/j.imlet.2021.03.004 33711331

[91] Kluck V Jansen T Janssen M Comarniceanu A Efde M Tengesdal IW. Dapansutrile, an oral selective NLRP3 inflammasome inhibitor, for treatment of gout flares: an open-label, dose-adaptive, proof-of-concept, phase 2a trial. Lancet Rheumatol. (2020) 2:e270–80. doi: 10.1016/S2665-9913(20)30065-5 PMC7523621 33005902

[92] Lonnemann N Hosseini S Marchetti C Skouras DB Stefanoni D D'Alessandro A. The NLRP3 inflammasome inhibitor OLT1177 rescues cognitive impairment in a mouse model of Alzheimer’s disease. Proc Natl Acad Sci U.S.A. (2020) 117:32145–54. doi: 10.1073/pnas.2009680117 PMC7749353 33257576

[93] Cho H Balaji S Hone NL Moles CM Sheikh AQ Crombleholme TM. Diabetic wound healing in a MMP9-/- mouse model. Wound Repair Regener. (2016) 24:829–40. doi: 10.1111/wrr.2016.24.issue-5 27292154

[94] Coll RC Hill JR Day CJ Zamoshnikova A Boucher D Massey NL. MCC950 directly targets the NLRP3 ATP-hydrolysis motif for inflammasome inhibition. Nat Chem Biol. (2019) 15:556–9. doi: 10.1038/s41589-019-0277-7 31086327

[95] Luciunaite A McManus RM Jankunec M Racz I Dansokho C Dalgediene I. Soluble Abeta oligomers and protofibrils induce NLRP3 inflammasome activation in microglia. J Neurochem. (2020) 155:650–61. doi: 10.1111/jnc.14945 31872431

[96] Torres S Brol MJ Magdaleno F Schierwagen R Uschner FE Klein S. The specific NLRP3 antagonist IFM-514 decreases fibrosis and inflammation in experimental murine non-alcoholic steatohepatitis. Front Mol Biosci. (2021) 8:715765. doi: 10.3389/fmolb.2021.715765 34513923 PMC8425476

[97] Wang X Sun K Zhou Y Wang H Zhou Y Liu S. NLRP3 inflammasome inhibitor CY-09 reduces hepatic steatosis in experimental NAFLD mice. Biochem Biophys Res Commun. (2021) 534:734–9. doi: 10.1016/j.bbrc.2020.11.009 33213837

[98] Madurka I Vishnevsky A Soriano JB Gans SJ Ore DJS Rendon A. DFV890: a new oral NLRP3 inhibitor-tested in an early phase 2a randomised clinical trial in patients with COVID-19 pneumonia and impaired respiratory function. Infection. (2023) 51:641–54. doi: 10.1007/s15010-022-01904-w PMC9473473 36104613

[99] Huang Y Jiang H Chen Y Wang X Yang Y Tao J. Tranilast directly targets NLRP3 to treat inflammasome-driven diseases. EMBO Mol Med. (2018) 10:e8689. doi: 10.15252/emmm.201708689 29531021 PMC5887903

[100] He H Jiang H Chen Y Ye J Wang A Wang C. Oridonin is a covalent NLRP3 inhibitor with strong anti-inflammasome activity. Nat Commun. (2018) 9:2550. doi: 10.1038/s41467-018-04947-6 29959312 PMC6026158

[101] McFarthing K Buff S Rafaloff G Fiske B Mursaleen L Fuest R. Parkinson’s disease drug therapies in the clinical trial pipeline: 2023 update. J Parkinsons Dis. (2023) 13:427–39. doi: 10.3233/JPD-239901 PMC10357160 37302040

[102] Coll RC Schroder K Pelegrin P. NLRP3 and pyroptosis blockers for treating inflammatory diseases. Trends Pharmacol Sci. (2022) 43:653–68. doi: 10.1016/j.tips.2022.04.003 35513901

[103] Deardorff WJ Grossberg GT. Targeting neuroinflammation in Alzheimer’s disease: evidence for NSAIDs and novel therapeutics. Expert Rev Neurother. (2017) 17:17–32. doi: 10.1080/14737175.2016.1200972 27293026

[104] Karkhah A Saadi M Pourabdolhossein F Saleki K Nouri HR. Indomethacin attenuates neuroinflammation and memory impairment in an STZ-induced model of Alzheimer’s like disease. Immunopharmacol Immunotoxicol. (2021) 43:758–66. doi: 10.1080/08923973.2021.1981374 34585992

[105] Feng X Hu J Zhan F Luo D Hua F Xu G. MicroRNA-138-5p regulates hippocampal neuroinflammation and cognitive impairment by NLRP3/caspase-1 signaling pathway in rats. J Inflammation Res. (2021) 14:1125–43. doi: 10.2147/JIR.S304461 PMC8009546 33814920

[106] Zhang M Wang L Huang S He X. MicroRNA-223 targets NLRP3 to relieve inflammation and alleviate spinal cord injury. Life Sci. (2020) 254:117796. doi: 10.1016/j.lfs.2020.117796 32417375

[107] Han C Guo L Yang Y Guan Q Shen H Sheng Y. Mechanism of microRNA-22 in regulating neuroinflammation in Alzheimer’s disease. Brain Behav. (2020) 10:e01627. doi: 10.1002/brb3.v10.6 32307887 PMC7303389

[108] Ali M Gupta M Wani A Sharma A Abdullaha M Kour D. IIIM-941, a stilbene derivative inhibits NLRP3 inflammasome activation by inducing autophagy. Front Pharmacol. (2021) 12:695712. doi: 10.3389/fphar.2021.695712 34248643 PMC8267097

[109] Cheng ZY Xia QP Hu YH Wang C He L. Dopamine D1 receptor agonist A-68930 ameliorates Abeta(1-42)-induced cognitive impairment and neuroinflammation in mice. Int Immunopharmacol. (2020) 88:106963. doi: 10.1016/j.intimp.2020.106963 33182028

[110] Liu X Hao W Qin Y Decker Y Wang X Burkart M. Long-term treatment with Ginkgo biloba extract EGb 761 improves symptoms and pathology in a transgenic mouse model of Alzheimer’s disease. Brain Behav Immun. (2015) 46:121–31. doi: 10.1016/j.bbi.2015.01.011 25637484

[111] Han X Xu T Fang Q Zhang H Yue L Hu G. Quercetin hinders microglial activation to alleviate neurotoxicity via the interplay between NLRP3 inflammasome and mitophagy. Redox Biol. (2021) 44:102010. doi: 10.1016/j.redox.2021.102010 34082381 PMC8182123

[112] Zhou F Lian W Yuan X Wang Z Xia C Yan Y. Cornuside alleviates cognitive impairments induced by Abeta(1-42) through attenuating NLRP3-mediated neurotoxicity by promoting mitophagy. Alzheimers Res Ther. (2025) 17:47. doi: 10.1186/s13195-025-01695-w 39972387 PMC11837312

[113] Li JY Chen MB Fu TT Liu FF Liu J Xu CJ. Discovery of structurally intriguing diterpenoids as anti-neuroinflammatory agents from mangrove plant Excoecaria agallocha L. via inhibiting macrophage polarization and inflammasome. Phytochemistry. (2025) 234:114440. doi: 10.1016/j.phytochem.2025.114440 39952580

[114] Zhang C Qiu WQ Yu L Pan R Teng JF Sang ZP. Development of cancer-associated fibroblasts subtype and prognostic model in gastric cancer and the landscape of tumor microenvironment. Int J Biochem Cell Biol. (2022) 152:106309. doi: 10.1016/j.biocel.2022.106309 36174922

[115] Qiu WQ Pan R Tang Y Zhou XG Wu JM Yu L. Lychee seed polyphenol inhibits Abeta-induced activation of NLRP3 inflammasome via the LRP1/AMPK mediated autophagy induction. BioMed Pharmacother. (2020) 130:110575. doi: 10.1016/j.biopha.2020.110575 32768883

[116] Li N Li M Guo M Meng T Mu B Yu J. Eriodictyol improves cognitive function in 5xFAD mice of Alzheimer’s disease by inhibiting the microglia NLRP3 inflammasome signaling pathway. Xi Bao Yu Fen Zi Mian Yi Xue Za Zhi. (2023) 39:973–80. 37980548

[117] Li L Li WJ Zheng XR Liu QL Du Q Lai YJ. Eriodictyol ameliorates cognitive dysfunction in APP/PS1 mice by inhibiting ferroptosis via vitamin D receptor-mediated Nrf2 activation. Mol Med. (2022) 28:11. doi: 10.1186/s10020-022-00442-3 35093024 PMC8800262

[118] Melchiorri D Merlo S Micallef B Borg J-J DrÃ¡fi FJFiP. Alzheimer’s disease and neuroinflammation: will new drugs in clinical trials pave the way to a multi-target therapy? Front Pharmacol. (2023) 14:1196413. doi: 10.3389/fphar.2023.1196413 37332353 PMC10272781

[119] Thawabteh AM Ghanem AW AbuMadi S Thaher D Jaghama W Karaman D. Recent advances in therapeutics for the treatment of alzheimer’s disease. Molecules. (2024) 29:5131. doi: 10.3390/molecules29215131 39519769 PMC11547905

